# Breaking down the barriers: Understanding migrant workers’ access to healthcare in Malaysia

**DOI:** 10.1371/journal.pone.0218669

**Published:** 2019-07-03

**Authors:** Tharani Loganathan, Deng Rui, Chiu-Wan Ng, Nicola Suyin Pocock

**Affiliations:** 1 Centre for Epidemiology and Evidence-based Practice, Department of Social and Preventive Medicine, University of Malaya, Kuala Lumpur, Malaysia; 2 School of Public Health, Kunming Medical University, Kunming, China; 3 United Nations University—International Institute for Global Health (UNU-IIGH), Kuala Lumpur, Malaysia; 4 Gender Violence & Health Centre, London School of Hygiene and Tropical Medicine, London, United Kingdom; Brown University, UNITED STATES

## Abstract

**Background:**

Malaysia is widely credited to have achieved universal health coverage for citizens. However, the accessibility of healthcare services to migrant workers is questionable. Recently, medical fees for foreigners at public facilities were substantially increased. Mandatory health insurance only covers public hospital admissions and excludes undocumented migrants. This study explores barriers to healthcare access faced by documented and undocumented migrant workers in Malaysia.

**Methods:**

We use qualitative data from 17 in-depth interviews conducted with key informants from civil society organisations, trade unions, academia, medical professionals, as well as migrant workers and their representatives. We interviewed doctors working in public hospitals and private clinics frequented by migrants. Data were analysed using thematic analysis.

**Results:**

We found that healthcare services in Malaysia are often inaccessible to migrant workers. Complex access barriers were identified, many beyond the control of the health sector. Major themes include affordability and financial constraints, the need for legal documents like valid passports and work permits, language barriers, discrimination and xenophobia, physical inaccessibility and employer-related barriers. Our study suggests that government mandated insurance for migrant workers is insufficient in view of the recent increase in medical fees. The perceived close working relationship between the ministries of health and immigration effectively excludes undocumented migrants from access to public healthcare facilities. Language barriers may affect the quality of care received by migrant workers, by inadvertently resulting in medical errors, while preventing them from giving truly informed consent.

**Conclusions:**

We propose instituting migrant-friendly health services at public facilities. We also suggest implementing a comprehensive health insurance to enable healthcare access and financial risk protection for all migrant workers. Non-health sector solutions include the formation of a multi-stakeholder migration management body towards a comprehensive national policy on labour migration which includes health.

## Introduction

The fundamental right of every human being to enjoy the highest achievable standard of health is enshrined in the Constitution of the World Health Organization [1]. The rights-based approached to health is reaffirmed with the 2030 Agenda for Sustainable Development, which emphasises universal health coverage and health equity, articulated in the concept of “leave no one behind” [2, 3].

Migrant workers are a vulnerable group, often exposed to poor living and working conditions, facing discrimination and social exclusion, and lacking the power to negotiate for healthcare in receiving countries [4]. Receiving countries often restrict access to healthcare services to regular, documented migrants, which has adverse implications for both individual and population health [5–7].

Malaysia is an upper middle-income country with a robust economy based on diverse, labour-intensive sectors. Malaysia is a net importer of foreign labour, with documented migrant workers consisting 15% of the labour force [8]. The Ministry of Home Affairs estimated 2.1 million regular, documented migrant workers in Malaysia in 2015 [9]. Due to their lack of legal status, irregular or undocumented migrants are invisible to policy-makers, thus their numbers remain contentious. Lee Hwok- Aun and Leng estimated a total of 3.85 to 5.3 million migrant workers in Malaysia in 2018, including undocumented workers [10]. At present, 15 countries supply labour to Malaysia, with the majority from Indonesia (39%), Nepal (24%), Bangladesh (13%) and Myanmar (7%). Migrants work in construction, agriculture and plantations, manufacturing, services and domestic work [9]. These are low-skill, low-wage jobs of less than MYR 2,000 (USD 465) per-month, unfavourable to citizens and crudely described as ‘Dirty’, ‘Difficult’ and ‘Dangerous’ (3Ds). Migrant workers are not to be confused with ‘expatriates’, a term used in Malaysia to denote foreigners employed in highly-skilled technical posts, middle- or top-managerial posts or professionals [11]. Despite being one of the largest migrant receiving countries in South East Asia [12, 13], Malaysia is not a signatory to the International Convention on the Protection of the Rights of All Migrant Workers and Members of Their Families [14]. Within the ASEAN region, only Indonesia and the Philippines, predominantly migrant sending countries, have ratified international treaties protecting the rights of migrant workers [15].

Malaysia has a mixed public-private healthcare system. The country is widely credited to have achieved universal health coverage through a tax-based public healthcare system, which guarantees access to care for citizens with minimal user fees. The private healthcare system is mainly for-profit and financed by fee-for-service [16, 17]. While public healthcare is highly subsidised for citizens, non-citizens are charged higher rates. Fees specific for non-citizens were first gazetted in 2003 and later substantially revised in 2014, to remove all elements of government subsidy [18–20]. Foreign nationals affected by this policy change include migrant workers, asylum-seekers, refugees, foreign students, foreign spouses, tourists and expatriates. Table 1 compares the medical charges for non-citizens with citizens.

**Table 1:** Charges for Malaysian citizens and non-citizens at public clinics and hospitals.

	Malaysian citizen	Non-citizen^2^	Price difference^3^
**Ward deposits**			
3rd Class (Medical)	MYR 20 (USD 4.6)	MYR 1,400 (USD 325.6)	70 X
3rd Class (Surgical)	MYR 30 (USD 7.0)	MYR 2,800 (USD 651.2)	93 X
**Daily ward charges**^1^			
3rd Class	MYR 3 (USD 0.7)	MYR 160 (USD 37.2)	53 X
**In-patient treatment charges**^1^			
3rd Class	Free	MYR 100 (USD 23.3)	+ 100
**Out-patient treatment charges**^1^			
Out-Patient Department	MYR 1 (USD 0.2)	MYR 40 (USD 9.3)	40 X
Specialist Clinic	MYR 5 (USD 1.2)	MYR 120 (USD 27.9)	24 X

NOTE. All medical charges are reported in Malaysian Ringgit (MYR) and United States Dollars (USD).

^1^ Treatment charges for non-citizens do not include investigation, procedure or medication

^2^ Exception is given to non-citizens with Permanent Residence status

^3^ Price differences for charges to non-citizens compared with Malaysian citizens

Source: Official Website of Hospital Kuala Lumpur. Ministry of Health [21]

Two government mandated insurance schemes are obligatory for the annual renewal of work permits of documented migrant workers: The Hospitalisation and Surgical Scheme for Foreign Workers (SPIKPA) and since 1 January 2019, the Employment Injury Scheme for Foreign Workers under the government’s Social Security Organization (EI-SOCSO). SPIKPA is a mandatory private medical insurance scheme which can be purchased from 25 insurance providers, which provides for annual coverage of MYR 10,000 (USD 2,325) for hospital admissions and surgery at Ministry of Health (MOH) hospitals, with an annual premium of MYR 120 (USD 28) per worker. The SPIKPA scheme is mandatory for migrant workers with premiums paid by either the employer or worker. The exemption being for domestic helpers and workers in plantations sectors where the insurance is optional, with employers allowed to opt-in by paying premiums [22]. EI-SOCSO is a social security scheme providing occupational illness or injury benefits in line with the SOCSO scheme for Malaysian citizens, with the exception of education benefits of enrolee’s dependents and physical rehabilitation benefits. The contribution rate is 1.25% of the insured migrant worker’s monthly wages, which is meant to be paid by the employer. Accidental death compensation is capped at RM 25,000, while permanent disablement benefit is based on 90% of the insured foreign worker’s average daily wage, subject to a minimum of RM30 and a maximum of RM118.50 per day [23, 24]. Repatriation and funeral benefits are also included. EI-SOCSO replaced the Foreign Worker’s Compensation Scheme (FWCS), which provided a maximum lump sum compensation payment of RM23,000 (USD 5,348) for accidental death, disablement or disease related to employment, which included compensation for repatriation, hospitalisation and medical expenses [25–28].

The Malaysian Immigration Act 1959 stipulates that all migrant workers must pass a pre-departure health test before being allowed to work in Malaysia. Documented migrant workers are also required to pass a pre-employment health examination within the first month of arrival, and are subsequently subject to mandatory annual health examinations as a condition for work permit renewals [29, 30]. In addition, some employers provide workers with medical treatment at private clinics, although the extent of this coverage varies.

Thus, migrant workers are expected to be healthy upon entry into Malaysia. However, living and working conditions, and exclusion from health services may predispose migrants to poor health. Access to healthcare goes beyond service availability, to utilisation as determined by financial, organisational, socio-cultural and other barriers that limit the use of healthcare, thereby affecting health outcomes [31, 32]. This study was motivated by the paucity of peer-reviewed publications on migrant worker’s access to healthcare in Malaysia [33]. We explored the barriers to accessing healthcare faced by both documented and undocumented migrant workers in Malaysia through a series of in-depth interviews with key stakeholders.

## Materials and methods

Qualitative methods were used in this study. This study is part of a larger project evaluating the policies protecting the health of migrant workers in Malaysia and China and returnees in Myanmar. Part of the data collected here will be presented in a companion paper on comparative policy analysis and has thus been excluded from the findings of this paper.

### Definition of terms

The International Labour Organization defines a migrant worker, as a person who migrates from one country to another with the view of being employed [34]. Regular or documented migrants are those employed in another country with the requisite legal documents, like valid passports and work permits. Irregular or undocumented migrants are those who enter a country in search of employment without the necessary legal documents, as well as those having entered the country legally, have either violated terms of their visa or over-stayed beyond the authorised period [35–37].

### Sampling and recruitment

Migrant workers in Malaysia are a heterogenous group. We aimed to capture the viewpoints of different stakeholders to better triangulate the barriers faced by migrant workers in accessing healthcare in Malaysia. We purposefully sampled key informants with expertise in migrant issues in Malaysia, including civil society organisations, international organisations, trade unions, academia, medical professionals as well as migrant workers and their representatives. Our initial sampling frame was formulated from the participant list of a 2017 expert workshop on Migrant and Refugee Health held in Kuala Lumpur, Malaysia [38]. We also contacted general practitioners at private medical clinics and medical doctors working at public hospitals. Potential participants were invited to participate by telephone and email and sent participant information sheets and consent forms. Further snowball sampling with interview participants was conducted until the researchers agreed that additional interviews would not yield new information, as theoretical saturation was reached.

A total of 17 in-depth interviews were conducted of 18 individuals (two participants were interviewed together) over a period of 3 months, from July to September 2018. All interviews were conducted in Kuala Lumpur and its surroundings, at locations chosen by the participants. All study participants were based in Malaysia and were either migrant workers or those working closely with migrant workers in case management, training and advocacy, legal aid, research or the provision of medical services.

Twelve participants were Malaysian. Of the 6 non-Malaysians interviewed, four identified themselves as migrant workers. Three of the 4 migrant workers interviewed were also members of civil society organisations or trade unions. We found that migrant workers organize themselves in groups based on country of origin rather than occupational groups (construction, manufacturing, plantation etc.). As such, we interviewed the main groups representing workers from Indonesia, Bangladesh, Nepal and Philippines. As domestic workers are a separate group with specific vulnerabilities, we interviewed representatives from their communities.

The immigration status of a migrant worker is highly sensitive, as it could result in detention and deportation. For ethical reasons, we did not specifically target undocumented migrants for interviews or probe participants on their immigration status. Migrant representatives and others interviewed discussed issues relevant to both documented and undocumented migrant workers in Malaysia.

The medical professionals interviewed were doctors working in public hospitals (emergency care, intensive care and general medicine wards) and private clinics providing outpatient services to migrant workers. Participants interviewed from international organisations were from an international agency and 2 civil society organisations with offices in Malaysia. Of those invited to participate, we received 7 negative responses, mostly from government officials and employers’ organisations. Some who declined participation expressed concern mostly on the inclusion of undocumented workers in the scope of this study. Table 2 describes the characteristics of the study participants.

**Table 2:** Characteristics of the study participants (n = 18).

**Nationality**	**No**
Malaysian	12
Non-Malaysian	
Migrant worker^1^	4
Non-migrant worker	2
**Total**	**18**
**Organisation type**	**No**
Local civil society organisations (CSO)	3
International organisations (IO)	3
Trade Unions (TU)	2
Medical doctors (MD)	4
Academia (AC)	2
Migrant worker^1^ (MW)	4
**Total**	**18**

^1^ Of the 4 migrant workers who were interviewed, only 1 identified himself as a worker only. The other 3 identified themselves as migrant representatives and were also members of civil society organisations (2) or trade unions (1).

### Ethics

The health and welfare of migrant workers is a topic of contention in Malaysia. We sought to minimise harm to study participants by assuring anonymity and confidentiality. Informed consent was obtained from each participant at recruitment. All participants agreed to be audio recorded and quoted anonymously in publications. Study participation was voluntary, and participants were informed that they could refuse to answer questions or terminate interviews at any point.

The lead author (TL) conducted all interviews. As a medical doctor and academic, we recognise that TL would likely be considered as being in a trusted authority position by participants, particularly with migrant workers. To minimize potential effects of social distance and power imbalances between the researcher and participants, the majority of interviews were conducted at a location of the participants’ choosing, in a space they were comfortable in and at a time convenient to them. Migrant participants, in particular, were assured that they could refuse to answer questions or to end the interview at any time. In doing so, we hoped that participants felt that they could exert a degree of control over the interview process.

This study was approved by the Medical Ethics Committee, University Malaya Medical Center and the Medical Research and Ethics Committee, Ministry of Health, Malaysia (Approval numbers: UM.TNC2/UMREC-238 and NMRR-18-1309-42043).

### Data collection and analysis

Semi-structured interview guides were developed to seek participants’ perspective on barriers to healthcare access for migrant workers in Malaysia. Questions were tailored towards the professional and organisational background of participants.

One researcher (TL) conducted all interviews in English and Bahasa Malaysia (also called the Malay language or Bahasa Melayu) and prepared field notes and impressions for all the interviews. Fourteen face-to-face interviews were conducted at locations chosen by the participants. Three interviews were conducted via telephone. The interviews averaged 52 minutes in length. Audio recordings were then transcribed verbatim. Concurrent data analysis was conducted to inform data collection and refine question guides. See S1 File for interview guides.

Data were analysed using thematic analysis [39]. Data analysis was conducted in an immersive, exploratory and inductive manner. Researchers listened to the audio recordings and edited transcripts for accuracy. Transcripts were then coded into emerging themes using NVivo 12 Pro software. Codes and themes were refined by repeated readings of all transcripts, field notes and impressions. Regular discussions helped to further refine coding and collapse codes into themes, in addition to giving attention to negative cases and minor themes. Interviews in Bahasa Malaysia were analysed in the same language, while extracted quotations were translated. Some interviewees spoke using broken English, necessitating literal translation for quotations.

All costs are reported in Malaysian Ringgit (MYR) and United States Dollars (USD), using the 2017 World Bank exchange rate of 4.30 [40].

## Results

Major barriers identified were financial barriers and affordability, lack of legal documentation, language barriers, discrimination and xenophobia, physical barriers and employer-related barriers. Table 3 summarises the main themes emerging from this study.

**Table 3:** Main findings of the study.

**Financial barrier and affordability**
• Migrant workers pay out-of-pocket for outpatient clinic visits • Health insurance is inadequate to cover the increase in medical fees at public hospitals • Migrant workers and employers are unaware of health insurance • Migrant workers financial constraints are a major barrier for healthcare access • Fear of high medical fees at public hospitals result in healthcare avoidance • Migrant workers’ ability to pay affects doctors’ management
**Undocumented migrants, legal status and health**
• Administrative requirements to check documents at public facilities limits healthcare access • Migrant workers prefer private clinics, as care is given without scrutinising documents • Undocumented workers may forge or falsify documents to seek care • Undocumented workers avoid necessary hospital care with unfortunate consequences
**Language barriers**
• Communication is a major problem faced by migrants and healthcare providers • The inability to communicate frustrates doctors sometimes resulting in brusque treatment • Language barriers may result in compromised patient safety from medication errors and poorly obtained consent for procedures • Owing to the lack of formal interpreters, doctors employ various strategies to overcome language barriers
**Discrimination and xenophobia**
• Xenophobia experienced by non-citizens are not specific to the healthcare setting • Perception of discrimination at public facilities due to the checking of documents at registration counters • Perception that medical doctors are negligent towards migrant workers may be due to poor communication
**Physical distance, freedom of movement and transport**
• Migrant workers fear of harassment by enforcement agents thus are unwilling to travel for treatment • Physical distance to healthcare facilities is a hindrance to rural plantation workers, while the lack of freedom of movement is a barrier for domestic helpers
**Employer-related barriers**
• Migrant workers are not provided paid sick leave • Migrant workers need permission to seek treatment • Employers withhold migrant workers’ passports

### Financial barriers and affordability

#### Migrants pay out-of-pocket for outpatient clinic visits

All participants agreed that affordability is of critical concern to migrant workers when accessing healthcare. The SPIKPA insurance scheme only provides coverage for hospitalisation and surgery at MOH hospitals. Most employers do not pay for the healthcare of workers, as such workers’ pay out-of-pocket (OOP) for outpatient visits.

Only larger, multinational companies may provide basic medical care through in-house health services or panel clinics. Panel clinics in Malaysia are one or more private clinics assigned by employers to provide outpatient treatment for their employees. Nonetheless, participants affirmed that most businesses in Malaysia are small- and medium- enterprise (SME) companies that do not provide healthcare to migrant workers.

“*By and large*, *we see that for migrant workers*, *the company doesn’t cover [healthcare costs]*. *This is very predominant in SMEs*. *Big companies*, *they may cover the worker’s medical*. *Suppose the workers have the flu [*…*]*, *he has to fork out the money to go for outpatient treatment*.*” CSO-2*

Participants shared that some employers may initially pay for clinic visits, then later deduct the healthcare payments from the worker’s salary.

“*We see a deduction of wages*, *[…] where the worker doesn’t have money*, *and he takes an advance from the employer to pay for the medical [bills]*. *That becomes a deduction of the wages*. *Because the company policy doesn’t cover their [medical] cost*.*” CSO-2*

Participants explained that the OOP charges for clinic visits are unaffordable to most migrant workers. Most migrant workers earn a minimum wage of MYR 1,000 (USD 233) per month [41], which they supplement by working overtime. This participant compared the cost of an average outpatient visit with weekly food expenses.

“*Migrant workers cannot afford to pay for medical bills […]*. *The bill is high*. *Yes*, *MYR 40 (USD 9) is high for them*. *One day’s salary*, *man*. *Just imagine*, *they can eat food for almost a week*, *you know*? *They spend about MYR 6 to MYR 7 (USD 1 to USD 2) only [on food] per-day*.*” TU-1*

#### Inadequate insurance coverage

Participants felt that the SPIKPA insurance scheme was inadequate in compensating foreigners’ high healthcare costs. This participant reported that although migrant workers are given life-saving treatment when they present at the emergency department, payment deposits required for hospital admission is often unaffordable for migrant workers.

"*The government hospitals will give them treatment [for] emergency cases*, *like an injury*. *But*, *they must pay some ‘guarantee’ money [deposit before admission]*. *The deposit will be higher*, *in some cases*, *it is MYR 3*,*000 (USD 698) before admitted into the hospital*. *That is very high*, *very high*.*” MW-1*

Participants reported that employers, knowing that insurance coverage is inadequate, often send migrant workers back to their country of origin with minimal medical care, as they don’t want to pay for additional, often definitive treatment.

“*[Employers are] sending them [migrant workers] back*, *sometimes without treatment*, *even though they are documented*. *That is because they would have gone for the initial treatment*, *[…] but follow up treatment*, *they can’t afford*. *Because the insurance would have finished already by then*. *So*, *what they do a lot is*, *they send the workers home*.*” IO-2*

#### Unaware of insurance provision

Participants explained that although documented workers’ pay for the mandatory SPIKPA insurance, many are unaware that the salary deductions are for medical insurance.

“*Every migrant worker*, *[…] has to pay for health insurance*. *[Its] deducted from their levy when they renew their permit*. *But*, *many workers have bad knowledge about that*. *So*, *they pay health insurance*, *[but] they don’t know how to claim their health insurance*.*” TU-2*

Participants revealed that migrant workers are not given an insurance card and are seldom provided with knowledge on the health benefits available to them.

" *[*…*] but the worker doesn’t have it [insurance card]*. *The worker doesn’t have the physical evidence or knowledge on that*.*" CSO-1*

One participant reported that even employers themselves may be unaware of insurance provision, as many leave the renewal of work permits to third-party agencies.

“*It is mandatory that they have [insurance]*. *But*, *sometimes*, *the employers just pass to the agent to arrange [everything]*, *like the working permit*. *[…] Sometimes*, *the employers also don’t know*. *Because they asked the agent to do all these things*.*” MW-1*

#### Migrant workers financial constraints

Because migrant workers are often paid a daily wage, they are not paid when they miss work. Loss of wages is an additional financial burden on workers, preventing them from accessing care. Participants spoke of migrant workers not being given time off work to seek treatment and being penalised with fines for non-attendance. One participant shared that workers stand to lose an attendance bonus if they miss work.

“*If they fail to attend*, *they also got deducted from the attendance bonus*. *Because the worker in the manufacturing environment*, *they are supposed to achieve certain attendance to earn the attendance bonus*.*” IO-1*

Migrant workers work hard and save their wages, to send home to support their families. Participants attested to the migrant worker’s tendency to prioritise remittance of income over all else, including healthcare and food.

“*I wouldn’t want my salary to be touched because I want to send the money back for my children’s education*. *So*, *if you are in that person’s shoe*, *you wouldn’t want to be them*. *Because you know there are mouths to feed*, *back in your country*. *You’d rather just sacrifice yours […]*. *Silently*.*” CSO-3*

Participants shared that most migrants pay for clinic visits themselves. Family, friends and community members may pool their resources and contribute to the payment of large hospital bills.

“*[*…*] those simple symptoms like flu […]*, *they pay from their own pocket*. *And for those hospital bills [*…*]*. *For the deposit*, *normally*, *the immediate family would help*. *If the amount shoots up to be so high*, *they would conduct fundraising among their community*.*” IO-1*

#### Fear of healthcare fees lead to healthcare avoidance

Participants described migrant workers as less willing to spend money on healthcare. They delay or avoid seeking care by self-treating or using traditional medicine. Overall, most participants viewed traditional medicine as a cheaper alternative to conventional treatment, rather than a choice made due to cultural preference. Traditional medicine is also one of the few healthcare options available to the undocumented migrant.

"*[…] The traditional doctors who come from the country of origin*, *they give traditional medicine to the workers*. *[*…*] They may give a placebo*, *sugar water*, *[*…*] or ‘jamu’ [traditional herbs] to workers*. *It is like an alternative healthcare system for workers*. *So*, *if you can’t afford high fees in the clinic*, *then they have access to these people*, *especially if you are undocumented*.*" CSO-1*

Because of exorbitant OOPs, participants shared that migrant workers avoid treatment, often presenting late and in critical condition. Participants felt that poor outcomes may have been prevented if the workers sought medical treatment earlier.

“*That group that don’t have any [health] coverage*, *they avoid coming [to the clinic] because they can’t afford it*. *They know they don’t have enough money*, *then they try to wait*, *to prolong and then come a bit later*. *And then sometimes*, *the problem might worsen*, *and [*…*] it costs more to solve it then actually solving it at the beginning*.*” MD-2*

Differential pricing for foreigners and high OOP payments at public facilities was perceived as unfair.

"*We also fear going to the [public] hospital or clinic*. *At hospitals*, *there is a double charge*. *For example*, *if local pay MYR 100 (USD 23)*, *we pay MYR 200 (USD 47)*. *If local pay MYR 500 (USD 116)*, *the foreign worker pays MYR 1000 (USD 233)*. *So expensive*. *Double the cost*.*" MW-4 (translated from Malay)*

Participants expressed that the fear of exorbitant hospital charges was the main reason given by migrant workers for avoiding public facilities, other than the requirement of legal documents.

#### Ability to pay affects doctors management

Medical doctors interviewed shared that in the case of migrant workers, doctors are often forced to make management decisions based on the patient’s ability to pay. Resource constraints compromise the doctor’s ability to provide the best clinical care as needed.

“*I have seen patients in the worst-case scenario*, *[…]*, *maybe requiring intubation*, *requiring CT scan*, *or very high dose antibiotics*, *which are expensive*. *And*, *each time we want to consider treatment*, *we would have to consider who would pay for this treatment*. *So*, *there is a lot of delay in a lot of cases*. *Of course*, *the patient’s welfare will be compromised because our hands are tied*.*” MD-3*

### Undocumented migrants, legal status and healthcare

#### Access to healthcare is linked to immigration status

Participants revealed that the lack of legal documents, like valid passports and work permits, are a major barrier to accessing care at public facilities. Patients are routinely asked to produce these documents at the registration counters of public facilities. Healthcare workers are obliged to inform the police or the immigration department when they encounter undocumented migrants. Some public hospitals have police and immigration counters located on their premises. Hence, undocumented migrants are detained and taken to immigration detention centres after receipt of emergency treatment. Participants declared that the perceived close cooperation between the health ministry and the immigration department essentially denies undocumented workers healthcare access at public hospitals.

“*[Migrant workers] need documents at the public clinics*. *Without documents*, *they don’t treat you*. *And now*, *most [public] hospitals have Immigration Desks inside*. *Because they say the ‘undocumented’ will come and then after they seek treatment*, *they run off*. *When the person who is ‘undocumented’ comes to see [the doctor]*, *they will refer them to the Immigration Desk*.*” CSO-2*

Despite mandatory reporting requirements, participants agreed that most medical doctors in public hospitals provided necessary emergency care irrespective of the patient’s immigration status or ability to pay.

“*We just resuscitate*. *Whatever we think we need to do we just do first*, *regardless of whatever documentation or payment*. *After the emergency treatment is given then later the hospital admin [administration] will deal with it [*…*]” MD-4*

#### Private clinics do not check documents

Overall, most participants explained that migrant workers prefer to use private clinics for basic medical care. Private clinics generally treat all patients without discriminating based on immigration status.

“*At private clinics*, *they don’t ask if you have a [work] permit or you don’t have a permit*. *In government clinics*, *they definitely will see if you have a permit or not*.*” TU-2*

This participant also said that doctors in the private sector do not report undocumented patients because it is not their role. He emphasised that the role of the doctor is to treat patients.

‴*We [doctors in private clinics] do not report them to immigration […]*. *It is only treatment*. *That is our part*. *So*, *the medical part is the only thing we handle*. *We don’t handle immigration issues at all*.*" MD-2*

Some interviewed described doctors treating undocumented patients as taking a professional risk. Others explained that doctors at private facilities are often reluctant to treat undocumented workers in emergencies.

“*It’s also dangerous you know*. *You as a practising doctor*, *you treat the person*, *and then the person died*. *How are you going to be accountable for it*? *Because he or she has got no documents*, *you are putting yourself and your profession in jeopardy*. *Some would say*, *'No*! *It’s my rice bowl*, *I wouldn’t want to'*.*” CSO-3*

#### Undocumented workers may falsify documents to seek care

Participants shared that undocumented workers in desperation may resort to fraud, falsifying documents to obtain needed care.

“*In a situation where medical attention is needed*, *but he or she doesn’t have documentation*, *[…] so they just photostat the passport*, *but the photo they change*. *So*, *it’s a different person*, *in order to access*. *If you are in their situation*, *you would do anything creative to seek medical attention*.*” CSO-3*

#### Undocumented workers avoid hospital care

Participants shared that undocumented migrants are likely to avoid necessary hospital care and are often only brought in to hospital either unconscious or critically ill. Participants also spoke of the consequences of not seeking treatment. This participant’s account illustrates unnecessary deaths caused by healthcare avoidance.

“*And some of them that are undocumented*, *they normally [*…*] just died in their workplace*. *They can’t seek treatment*. *They just stay in the sawmill*, *plantation [*…*] that they are in*. *They would just go off [die]*. *Just like that*.*” CSO-3*

### Language barriers

#### Inability to communicate is a major obstacle

Most participants maintained that language and communication is a major obstacle faced by both migrant workers and healthcare providers. Migrant workers in Malaysia come from different Asian countries. Participants spoke about the difficulties faced by the different nationalities.

“*Language also is a problem*. *For Indonesian*, *they have*, *I think*, *not a lot of problems*. *But*, *other countries like Bangladeshi*, *Myanmar*, *they have a lot of difficulties when meeting with the local doctor*.*” CSO-1*

One participant explained that even Indonesian nationals may have difficulty communicating with Malaysian doctors. Even though the Indonesian national language (Bahasa Indonesia) is similar to Bahasa Malaysia, not all Indonesians are conversant in Bahasa Indonesia. Also, subtle differences between Bahasa Malaysia and Bahasa Indonesia could easily result in misinterpretation.

“*[The Indonesians]*, *they also have a barrier of the language to describe their situation*. *Of course*, *when we talk about those people from Java and Jakarta*, *they are very fluent in Bahasa Indonesia*. *But*, *Indonesia*, *is a very diverse country*. *They are more than 250 languages spoken*. *[*…*] We always assume Bahasa Malaysia and Bahasa Indonesia is the same*, *but they are not*.*” IO-1*

#### Language barriers affect doctor’s treatment of migrant workers

One participant shared that the inability to communicate often frustrates medical professionals, resulting in impatience and sometimes brusque treatment of migrant workers.

“*[…] the terms that he or she use*, *may not be what you [the medical doctor] think it is*. *That is also another hindrance*. *You get so irritated*. *You start scolding him or her*. *And then*, *he [migrant worker] starts having a phobia*, *‘Waa*, *I come to seek help but now I get a scolding*. *Next round when I get sick*, *I won’t come again’*.*” CSO-3*

#### Language barriers lead to medication errors

As described by participants, communication difficulties may be hazardous. This participant revealed that migrants often complain of perceived medication errors.

“*I went to the clinic the other day because I had fever*. *I told the doctor I had fever*. *The doctor gave me an injection*, *after that I could not walk for 5 hours*! *After that*, *I went back to the clinic and confronted the doctor*. *He apologised and admitted he gave me the wrong medication*. *[…] A lot of migrants have gone to the hospital*, *and the doctor gives the wrong medication*. *This is what happens when you can’t speak the language*. *Migrants always complain [of this]*. *Sometimes it’s very serious*. *Someone could die*.*” MW-4 (translated from Malay)*

While migrants themselves may be incapable of detecting medication errors, they sometimes perceive treatment to be ineffective or harmful. Participants expressed concern as doctors are unable to properly explain to migrants about their illness or give appropriate advice on treatment. Miscommunication and errors have resulted in the migrant workers’ mistrust of healthcare professionals.

“*Sometimes I have a headache*, *but the doctor gives me medication for stomach pain*. *[*…*] I think the doctor doesn’t understand our Bahasa [language]*. *When we are sick the doctor does not explain what’s wrong with us*. *We don’t know what the doctor is thinking […]*. *But he still gives us medicine*.*” MW-4 (translated from Malay)*

#### Language barriers lead to lack of consent

The language barrier restricts a doctor’s ability to properly explain procedures and obtain informed consent, resulting in misunderstanding, distress and migrant workers’ mistrust of medical professionals.

“*The injured worker said he explained many times to his employer and the doctor [that he did not want amputation]*. *But they didn’t understand him*. *He doesn’t know why they still amputated his arm […]” TU-2 (translated from Malay)*

The medical doctors interviewed expressed disbelief when told of migrant workers’ accounts of unauthorised procedures. Those interviewed denied this sort of practice.

“*In my practice*, *I’ll explain properly*. *Because*, *even though it is a verbal consent*, *I’ll make sure that at least the patient [can understand]*. *If the patient cannot understand*, *the family members or the employer understands the consent*. *Because it’s very important*. *Because they may sue us later*, *we don’t know […]” MD-4*

#### Strategies to overcome language barriers

The doctors interviewed also felt that communication with the migrant worker is a major problem. Some migrant workers bring companions, who act as informal interpreters. Doctors also attempt to find informal interpreters among their staff. As healthcare services in Malaysia do not provide formal interpreters, medical doctors are forced to be inventive, using Google Translate, sign language or even mimes to communicate with foreign patients.

“*[Communication] is very*, *very difficult*. *It's literally sign language and acting out the illness*, *and all sorts of things*. *If they bring a friend who can speak either English or Malay*, *then they become the translator [*…*]*. *If they come alone or they bring another friend*, *[who] also don’t know anything about language*, *and then we find it very tough*. *Literally*, *it’s sign language*. *Now we even [use] Google Translate*.*” MD-2*

Medical doctors often rely on Google Translate, but this is not always the best option. This doctor described that illiterate workers and those from rural areas, find the language spoken on Google Translate unfamiliar.

“*Some of them cannot read*, *so we have to use the voice [a function of Google Translate]*. *Sometimes the sound they can't understand*. *These fellows might be from the outskirts*, *with a different dialect*. *They won’t be able to understand*.*” MD-2*

Doctors interviewed shared their attempts to learn key phrases in various Asian languages to better converse with foreign patients.

"*Over the years*, *I have picked up a few words here and there in various languages*, *so I can conduct my history taking and examination*.*"MD-1*

Medical doctors at hospitals attempt to contact co-workers or employers to obtain a better history. Participants shared that they sometimes need to contact the police to find employers. Because of language barriers, doctors are forced to treat patients based on clinical presentation, without adequate history-taking.

“*Some of them*, *if they are legal*, *they will have their passport or their identity card*. *We will be able to either track them down through immigration or through the police if we are unable to get a phone number*. *If they do have a phone number*, *we will try to call to see whether the employer would be contactable*. *[…] the employers might not answer a phone call coming from the hospital*. *A lot of times*, *we get excuses that they are too busy to come and attend to their worker*.*” MD-3*

One participant expressed frustration that employers seldom sends anyone to accompany workers when they seek treatment. Participants expressed that if workers were accompanied by a local or someone familiar with the language, they would have fewer problems communicating with health staff.

### Discrimination and xenophobia

#### Xenophobia is not specific to the healthcare setting

One participant spoke of the animosity experienced by non-citizens everywhere in Malaysia. In her opinion, the healthcare setting is no different than others, in its treatment of non-citizens.

"*[Discrimination] actually is not only related to healthcare*. *We see xenophobia everywhere*. *When we go to the shopping market*, *any place where locals meet us*, *they act like we are from another planet*. *Like we are dirty or something like that*. *This is the real situation*.*" MW-1*

One participant shared that migrant workers are sensitive to the way locals address them and speak to them. According to this participant, most Malaysian’s are unaware that certain words used regularly may be construed by migrants as derogatory.

“*[…] many of us do not understand the sentiment*. *When you call them ‘Indon’*, *it is a humiliating salutation*. *Just like you call a Bangladeshi*, *as ‘Bangla’*. *For them*, *if you call them ‘Indon’*, *it is not an appropriate salutation for them*. *Of course*, *our people are used to it*, *even their people also call themselves ‘Indon’*. *But those educated Indonesian*, *they take it as the stigma of humiliation*.*” IO-1*

#### Perception that checking documents at hospitals is discriminatory

Front-line hospital workers are generally tasked with examining the workers’ documents, an administrative process which migrants fear. One participant described the scrutiny of documents as a discrimination faced by migrant workers at the healthcare settings.

“*[…] after the nurse registers*, *they will report to the police*. *Police are on duty there*. *They will check on their screen*, *[to see] whether they are documented and undocumented*. *[*…*] This is actually one of the discriminations they are facing*.*” CSO-1*

#### Perception that medical doctors are negligent towards migrant workers

One migrant worker interviewed articulated that Malaysian doctors treat migrant workers as if they were not of any consequence. This he felt was unfair, as workers’ pay hard-earned money and encounter many difficulties when seeking treatment.

“*I don’t always understand why*. *[…] I think Malaysian doctors look at migrants like [they don’t matter]*. *If sick or any problem also he doesn’t care*. *It doesn’t matter […]*. *But this is wrong*.*” MW-4 (translated from Malay)*

Others interviewed felt that the failure of doctors to take migrant worker’s health concerns seriously may be attributed to their inability to communicate.

### Physical barriers

#### Fear of harassment by authorities

Many participants expressed concern about the difficulties faced by migrant workers while travelling. According to those interviewed, both documented and undocumented migrant workers are frequently stopped by enforcement agents like the police, immigration or RELA, a paramilitary civil volunteers corps, with demands to inspect documents. This participant described migrant workers’ fear of harassment by the police:

"*Police his duty*, *he can anytime check*. *They will do the roadblock*. *[We] go anywhere with motorbike also*, *police can check [*…*]*. *We are migrants*, *he can check us*. *It is his duty what*? *[…] But*, *don't disturb*, *don't beat*, *don't catch*. *[…] People with permit also he wants to take money*. *He says*, *'Next time I'll catch you and send you to prison'*. *All sorts of threats*. *Of course*, *we're afraid*. *But isn't that the law*? *We also follow the law*. *If they are undocumented workers*, *can catch […]*. *But*, *the rest of us with permits*, *why do the police catch us*?*" MW-4 (Translated from Malay)*

One participant asserted that migrant workers are unwilling to travel far for treatment, preferring to pay for treatment at private clinics close to their living quarters, rather than travel to panel or public clinics located further away.

"*[Migrant workers] They would prefer to go somewhere which is close to their hostel*. *They don’t trust public transportation*. *Even when they are taking a taxi*, *they will get caught by enforcement agencies*. *And shockingly*, *several of them were caught with documents*. *All paid money*.*” TU-1*

#### Physical distance, freedom of movement and transport

Participants shared that physical distance was more a concern for those working in rural areas than those in urban areas. Participants spoke of the great geographical distances to travel from rural plantations to towns and healthcare facilities. Some plantations are particularly inaccessible, especially if employers do not provide transportation. Journeys may involve several hours by foot, road transport or even travel by boat. Participants acknowledged that big plantations often provide some healthcare facilities. However, these services may be hard to reach, because of the physical distance.

“*[…] Sometimes with small-holders*, *they don’t bother about this [providing healthcare]*. *For the big plantation*, *of course*, *they do have healthcare*. *But when the plantation is so big*, *for example in Sabah you can see 30*,*000 [or] 40*,*000 hectares of plantation [*…*]*. *Maybe the service is somewhere nearest to the main road*. *How about the ones inside [those in the interior]*? *Healthcare coverage is still a big question mark*.*” IO-1*

In urban areas, public transportation is easily available. However, participants spoke of travelling expenses, as an additional burden to healthcare costs.

“*[*…*] I walk and go*, *sometime taxi and go*. *[But] taxi is expensive*. *Sometimes*, *I pay MYR 5 (USD 1*.*2) or MYR 6 (USD 1*.*4) like that*. *And this is just for flu*, *cough [*…*]” MW-2*

Lack of freedom of movement is a concern for domestic helpers. Domestic helpers in Malaysia are generally not given off days or allowed to leave their workplace, which is their employer's house. This situation forces them to be dependent on their employers for all things, including healthcare.

“*[Domestic helpers] we cannot go to the hospital*, *we cannot even leave the house*. *We cannot even buy the tablet [medication]*. *[…] Asking from the employer only*.*” MW-3*

### Employer-related barriers and constraints

#### No paid sick leave

Participants acknowledged that most employers do not honour medical certificates (MC) issued by doctors, forcing workers to work even when sick. According to participants, employers often believe that workers are faking illness, and as such do not provide paid sick leave. This participant alluded to the employer’s focus on productivity, lacking concern for the worker's well-being.

“*[…] for many workers*, *if they go for the medical treatment*, *they don’t get an MC*. *Their salary will be deducted*, *and they will be fined from their attendance [bonus]*. *This happened because their [employer] said that they are not disciplined enough*. *Because the Malaysian employer is like*, *‘Every single dollar and cents counts’*. *So*, *for them*, *this is another input of the productivity*, *the human resources input*.*” IO-1*

This participant’s account illustrates the migrant workers’ vulnerability, as they feel they have no avenue to challenge unfair practices.

“*I think the employer thinks migrants are healthy*. *He doesn’t believe us*. *Does not think it’s serious*. *We can’t take any action*. *What can we do*? *If you don’t want to help us*, *why don’t you let us go ourselves [to the clinic]*? *But we can’t say this*. *They threaten us with suspension […]*.*” MW-4 (translated from Malay)*

Participants also reported that employers may compel medical doctors to restrict the issuance of MCs and the prescription of more expensive medication. This practice is especially prevalent among in-house doctors or those at panel clinics provided for workers by employers. Some participants shared that employers are often reluctant to allow migrant workers full access to healthcare, as they are concerned about misuse.

“*a lot of companies don’t want to give full [healthcare] access to foreigners*, *because they are worried of misuse*. *And you’ll be surprised some of them [migrant workers] would just come and visit you frequently*, *as either they seek MC*, *or you know their aches and pains*. *Some of them do really hard work […] and they tend to have body aches*.*” MD-2*

Participants also shared that undocumented workers are particularly vulnerable to exploitation by employers as they lack avenue for redress. Undocumented workers are thus unlikely to receive healthcare benefits or paid sick leave.

“*Some employers*, *they don’t pay because they say he is undocumented*. *So*, *they think the workers don’t have rights and don’t have access to report*. *Because if they [undocumented worker] report*, *definitely they will be sent to the jail*.*” MW-1*

#### Permission to seek treatment

One participant explained that some companies require migrant workers to obtain permission from supervisors to seek treatment.

“*The good ones [employers]*, *actually they have a panel system with the clinic*. *[…] They also have a slip system where they [migrant workers] have to go to the HR [human resources] to collect the slip*, *and then they will come to the clinic and we [doctors] see them and sign them off*. *So*, *they don’t pay anything*, *because we will use the slip to claim” MD-2*

Participants spoke of the process of obtaining permission to seek treatment, which is particularly troublesome when the worker is ill after working hours or is sick in his living quarters. Participants felt that the requirement for permission was a measure used by employers to further restrict a migrant worker’s access to care.

#### Employer withholds passports

Participants reported that although the practice is in violation of the Passport Act 1964, most employers keep their worker's passports. Those interviewed explained that employers usually justify this practice by saying that passports are taken for safe-keeping and to prevent workers from running away. The withholding of passports places the migrant workers in a vulnerable position.

“*They’re afraid to go to government hospitals because they don’t have their passport with them*. *Without the documents*, *they feel like they can be arrested [*…*]*. *When I asked them*, *‘Why don’t you have documents*?*’ They say*, *‘The employers keep it with them*.*’ […]” AC-1*

Without passports, documented workers are considered de facto undocumented and are dependent on their employers to navigate the health system. Participants explained that these migrant workers, although technically documented, are subject to arrest by the police and other enforcement agents because they do not hold their documents.

## Discussion

We found that healthcare services are often unavailable to migrant workers in Malaysia due to financial constraints. Healthcare costs are seldom borne by employers and migrant workers mostly pay OOP for healthcare [42]. Previous studies in Malaysia and elsewhere support our findings that migrant workers prefer private clinics, utilising public clinics infrequently [42–45]. Not surprisingly, indirect and non-medical costs of utilising health, including the loss of wages and the cost of transportation, restrict access to healthcare services.

Our findings suggest that the SPIKPA insurance is inadequate in view of the recent increases in medical fees at public hospitals. Furthermore, if hospital bills exceed the MYR 10,000 (USD 2,325) insurance ceiling, migrants are obliged to pay the remainder. Workers with outstanding medical bills will not be allowed to renew their work permits upon expiry, while their employers are black-listed by the Immigration Department [22, 25, 28]. As employees are responsible for the payment of hospital bills, migrant workers often flee from hospitals before being officially discharged [46]. As demonstrated in our findings and in other settings, many employers send workers home, rather than face expensive hospital charges [47].

Additionally, we found that financial constraints are likely to influence the quality and provision of services. Healthcare workers interviewed alluded to the inevitable rationing of care due to financial limitations. Hence, a comprehensive insurance package for migrant workers that encompasses a full range of services (preventive, promotive, curative, rehabilitative) if implemented, would provide access to quality healthcare and financial risk protection, fulfilling goals for truly inclusive UHC [48]. We suggest that the MOH, Malaysia evaluate the current fee structure for non-citizens at public facilities to consider utilization patterns and unpaid bills among non-citizens. The SPIKPA insurance scheme should also be revised to commensurate with the increased fee structure for non-citizens at MOH facilities.

The need to present legal documents at healthcare facilities and the fear of deportation is a major deterrent for healthcare utilisation. We found that migrant workers feel discriminated against, especially when asked to produce documents on registration at healthcare facilities. Discrimination based on documentation status, experienced as a stigma by undocumented migrants has been demonstrated in various settings [5, 49, 50]. Undocumented migrants are particularly insecure at public facilities, as they are liable for arrest and detention. Doctors interviewed expressed concern that withholding care based on legal status is against the ethics of the medical profession. Nevertheless, the MOH, Malaysia directs all health professionals to report the presence of undocumented foreigners to the police, as required by the Immigration Act [51].

Currently, Thailand is the only ASEAN member country, and among the few globally, that allows for undocumented migrants to opt-in to the national health insurance scheme, allowing for population-wide access to healthcare not restricted by citizenship [48, 52, 53]. Under the Thai Health Insurance Card Scheme (HICS), undocumented migrant workers can register at One Stop Service Centres (OSSCs) comprised of the Ministry of the Interior issuing temporary identity cards, the Ministry of Labour issuing temporary work permits, and the Ministry of Public Health conducting the medical exam and enrolling migrants in the HICS, with durations of 6 months to one year. Migrant workers must then undergo a Nationality Verification (NV) process at their home country embassy to receive full work permits and identity documents beyond this period. While the challenge remains that not all migrants undergo the NV process and therefore become totally undocumented again, the Thai system remains a rare example of a destination country which permits undocumented migrants to enrol in a national health insurance scheme [54]. Malaysia, as a major destination country for migrant workers, has some provision of care for documented migrants. Unfortunately, UHC in Malaysia is clearly restricted to citizens, with a higher fee system for foreigners and the exclusion of undocumented migrants from public healthcare services.

Poor communication affects the quality of care received. Those with limited local language proficiency report less satisfaction with care and poor understanding of their medical situation. Limited language proficiency also affects patient safety in terms of medical errors on the part of the prescribing physician, and adverse medication reactions resulting from patient’s misunderstanding of instructions [4, 55, 56]. Achieving sufficient patient understanding to assure informed consent is particularly challenging for patients without proficiency in local languages [57]. Our study suggests that translation provided by informal interpreters like family members, friends, or untrained staff members may be suboptimal, and reliance on informal interpreters may compromise clinical care. The MOH, Malaysia should consider developing a professional interpreter service at major hospitals in Malaysia. While providing trained, professional interpreters may not be feasible due to the complexity and expense of catering to the myriad of nationalities working in Malaysia, the health system should aspire towards establishing migrant-friendly services. A successful example is Thailand’s migrant-friendly services, which involved training voluntary community health workers from migrant communities as intercultural mediators and patient navigators, as well as providing health education material in migrant languages [52, 58, 59]. Cultural competence training and professional development opportunities such as mentoring and supervision of the health workforce are necessary approaches in other countries to combat issues of racism and discrimination experienced by marginalised communities [60].

Described here are complex barriers to healthcare access that goes beyond the health sector, involving labour and immigration policies. According to the Employment Act 1955, an employee shall be granted paid sick leave after an examination by a registered medical officer at the expense of the employer. Unfortunately, the act does not explicitly outline an employer’s responsibility to provide medical attention to employees [61], exacerbating financial barriers faced by migrant workers accessing much needed care. We suggest that the Employment Act 1955 be amended to ensure the employer’s payment for treatment at outpatient clinics, provision of paid sick leave and transportation to healthcare facilities. We suggest here that regular labour inspections be conducted to ensure the welfare of migrant workers, including the enforcement of Passport Act 1964 to prevent employer’s withholding of passports.

Migration policies in Malaysia are overwhelming focused on controlling migrant workers [62, 63], as reflected in our findings on immigration notification at health facilities. We suggest establishing a multi-stakeholder migration management body, that can work towards developing comprehensive labour migration policies which include health. The recent decision to include migrant workers in Malaysia’s social security scheme (SOCSO) in line with International Labour Organization (ILO) recommendations are promising [64–66]. But it remains to be seen how this policy will be implemented and its benefits in improving healthcare access.

We found that all migrant workers in Malaysia faced financial difficulties when accessing healthcare. Not surprisingly, the main barrier experienced by undocumented migrants were the lack of documents, such as valid work permits or passports, making it impossible for them to seek treatment at public facilities. Also, the lack of documents makes safe travel to seek treatment challenging for undocumented migrants. Interestingly, we found that most documented workers are similarly vulnerable as their passports were unlawfully withheld by employers. Undocumented migrants are not covered by SPIKPA health insurance for healthcare costs at MOH hospitals. But this is irrelevant, as migrants are unable to access public healthcare without valid documents. Other barriers faced, like language, discrimination, and transportation are similar for both documented and undocumented migrants. Table 4 summarises the main policy recommendations discussed here.

**Table 4:** Policy recommendations to address barriers to migrant worker’s access to healthcare.

Barriers	Policy Recommendations
Financial barriers and affordability	• Consider increasing the maximum coverage of SPIKPA insurance (MOH) • Consider developing a comprehensive insurance package that covers a full range of healthcare services (preventive, promotive, curative, rehabilitative) (MOH) • Evaluate the fee structure for non-citizens at public facilities (MOH)
Undocumented migrants, legal status and health	• Evaluate policy on reporting patients without valid documents to immigration (MOH, Immigration Department) • Consider insurance coverage options for undocumented migrants (MOH)
Language barriers	• Consider the development of a professional interpreter service at major hospitals (MOH) • Train voluntary community health workers as intercultural mediators (MOH, CSOs) • Provide health education materials in major migrant languages at health facilities and worksites (MOH, MOHR, CSOs)
Discrimination and xenophobia	• Train and develop the health workforce to ensure a culturally competent health system (MOH)
Physical distance, freedom of movement and transport	• Develop mechanisms to ensure that employer’s provide transport to health facilities in remote areas (e.g. plantations) and/or payment of transport expenses for migrants seeking care (MOHR) • Develop mechanisms to ensure that employer’s provide time off for migrant workers, including domestic workers, to seek healthcare (MOHR)
Employer-related barriers	• Revise the Employment Act 1955 to ensure the employer’s responsibility in migrant workers healthcare provision, including payment for care at outpatient visits, provision of paid sick leave and transportation to healthcare facilities (MOHR) • Enforce the Passport Act 1964 to ensure employers do not withhold worker’s passports (MOHR)

Note: MOH, Ministry of Health; MOHR, Ministry of Human Resources; CSO, Civil Society Organisation.

This study has several limitations. Migrant workers in Malaysia are a heterogeneous group from different countries of origin, with different linguistic and socio-cultural backgrounds, and working in diverse industries. Also, migrant workers in East Malaysia may have specific barriers to healthcare not explored here. This diversity in migrant populations results in disparate health experiences and vulnerabilities. Also, undocumented migrants are a hidden population, who are difficult to contact and interview. Nonetheless, by interviewing distinct groups including migrant workers and their representatives, civil society organisations, trade unions, academia and medical doctors, we were able to triangulate findings and obtain a broad understanding of the healthcare barriers faced by migrant workers in Malaysia. While the qualitative nature of this study precludes the generalisation of findings, we gained insight into the obstacles faced by migrant workers seeking care from multiple perspectives.

This study has several strengths. To our knowledge, this is the first qualitative study of its kind to examine and provide a detailed understanding of barriers to healthcare access faced by migrant workers in Malaysia. Our findings address an important gap in understanding of migrant health in Malaysia. We have suggested areas for future research and policy change, including a “health in all policies” approach to labour and immigration policies, expanded health insurance coverage to include preventive and rehabilitative services, and suggestions to make healthcare services more migrant-friendly.

## Conclusion

Despite achieving UHC for citizens, healthcare services in Malaysia are often inaccessible to migrant workers. Excluding migrants from national health provisions violates human rights and undermines national public health goals. A rights-based approach to health, integrating migrant workers health needs within national health strategies, will benefit the entire population. Ultimately, changes in migrant health-related policy will only come from political will. We urge Malaysia’s new government to recognize the immense contribution of migrant workers and to no longer continue policies of benign neglect, by testing and implementing policies that ensure no one, including migrant workers, are left behind.

## Supporting information

S1 File Interview guide. (DOCX)
