# Supplementary material for: Breaking down the barriers: Understanding migrant workers’ access to healthcare in Malaysia
Source: PLoS One. 2019 Jul 3;14(7):e0218669. doi: 10.1371/journal.pone.0218669 (PMC6608924; doi:10.1371/journal.pone.0218669)
Supplement: S1 File — (DOCX) [file pone.0218669.s001.docx]

Supplementary Information

# Interview Guide

## Migrant workers

Interview topics and questions that form the broad framework of discussion with migrant workers will include:

**Topics:**

- Knowledge and perception of healthcare services available
- Experience with access to healthcare in Malaysia
- Experience with barriers to access to healthcare in Malaysia
- Experiences with employers in relation to illness or injury
- Experience with healthcare workers with regards to healthcare treatment
- Suggestion for improvement in health policy or services available for migrants

**Introductory questions:**

1. Sex (M/F)
2. Date of Birth
3. Nationality
4. Years in Malaysia
5. Which country did you leave to come here?
6. What do you work as?
7. Are you employed by an individual or a company?

**Open questions:**

1. What are the most common health problems that you or your friends have faced during your stay in Malaysia? (examples)
2. Can you tell me where you or your friends will go for healthcare services when you are ill in Malaysia?
3. Can you share with me what is the healthcare experience like for migrant workers in Malaysia?

Prompts: How? Availability? Experience? Case studies?

1. What do you think regarding to healthcare services in Malaysia?

Prompts: awareness of services available for migrants, insurance schemes, and injury compensation schemes

1. What are the difficulties you or your friends have faced in accessing care?

What are the key barriers for migrant workers in accessing care in Malaysia?

Prompts: Barriers from individual, health system, community/cultural, stigma, geographical, financial, immigration status, fear of deportation? Case studies?

1. How do you pay for health care services?

What are the financial barriers for migrant workers in accessing healthcare in Malaysia?

Prompts: Are you covered by any insurance scheme? Does your employer pay your medical bills? Can you afford to seek treatment? Does seeking healthcare cause you financial hardship? Case studies?

1. What are your experiences with your employer/employers in relation to access to healthcare for illness or injury?

What is the experience of migrant workers with employers with regards to healthcare treatment?

Prompts: Are employers supportive? Will pay be docked for non-attendance? Will the worker be fired? Will employer pay for healthcare? Case studies?

1. What is your experience with healthcare workers with regards to healthcare treatment?

Prompts: positive/negative? Are they friendly? Communication barrier? Cultural appropriateness? Stigma? Case studies?

1. What are your suggestions for the improvement in health policy or services available for migrants in Malaysia?

## Key Informant interviews: NGOs, migrant representatives, trade unions, academia etc.

Interview topics and questions that form the broad framework of discussion on policy protecting the health of migrants will include:

Topics:

- Knowledge of healthcare policy and services available in Malaysia
- Experience with migrant access to healthcare in Malaysia
- Perceptions or experience of barriers to migrant access to healthcare in Malaysia
- Experiences with employers of migrants in relation to work related illness or injury
- Experience of migrants with healthcare workers
- Suggestion for improvement in health policy or services available for migrants

**Introductory questions**

For representatives of migrant workers communities:

1. What is your role in your community/organisation?
2. What communities or nationalities does your organisation represent?
3. What is the demographic profile of migrants in your community?
   (age, sex, occupation, marital status)
4. What is immigration status of the communities that your organisation represents? (documented/undocumented, economic migrants, refugees, stateless people)

**Open questions**

1. Can you tell me about healthcare policy and services available for migrant workers in Malaysia?

Prompts: awareness of services available for migrants, insurance schemes, and injury compensation schemes

1. Could you please share experience of migrant access to healthcare in Malaysia?

What is the healthcare experience like for migrant workers in Malaysia?

Prompts: Where? How? Availability? Experience? Case studies?

1. Could you please share experience of barriers to access to healthcare of migrant workers in Malaysia?

What are the key barriers for migrant workers in accessing care in Malaysia?

Prompts: Barriers from individual, health system, community/cultural, stigma, geographical, financial, immigration status, fear of deportation? Case studies?

1. What are the perceived barriers to access to healthcare for migrant workers in Malaysia

What are the key barriers for migrant workers in accessing care in Malaysia?

Prompts: Barriers from individual, health system, community/cultural, stigma, geographical, financial, immigration status, fear of deportation? Case studies?

1. What kind of healthcare facilities do migrants go to when they are ill?

Prompts: Public or private? And why? Do many opt not to seek care?

1. How do migrants pay for healthcare?

What are the financial barriers for migrant workers in accessing healthcare in Malaysia?

Prompts: Do you know migrants covered by insurance schemes? Do employers pay for medical bills? Can migrants afford to seek treatment? Does seeking healthcare cause financial hardship? Case studies?

1. What are migrants experience with employers when they are ill?

What is the experience of migrant workers with employers with regards to healthcare treatment?

Prompts: Are employers supportive? Will pay be docked for non-attendance? Will the worker be fired? Will employer pay for healthcare? Case studies?

1. What are migrants experience with healthcare workers?

Prompts: positive/negative? Are they friendly? Communication barrier? Cultural appropriateness? Stigma? Case studies?

1. How the organization (you are representing) involved in migrant health issue? What is your organization’s role?

Prompts: What are the organization’s aims and activities relating migrant access to health care? What are the key success and challenges of your organization while working on migrant health issue? What is your opinion on collaboration among different sectors working on migrant health issue?

1. What are your suggestions to improve health policy and services for migrants in Malaysia?

## Medical doctors

Interview topics and questions that form the broad framework of discussion on policy protecting the health of migrants will include:

Topics:

- Knowledge of healthcare policy and services available in Malaysia
- Experience with migrant access to healthcare in Malaysia
- Perceptions or experience of barriers to migrant access to healthcare in Malaysia
- Experiences with employers of migrants in relation to work related illness or injury
- Experience of healthcare workers with migrants
- Suggestion for improvement in health policy or services available for migrants

**Introductory questions**

1. In what capacity do you deal with migrant workers?
2. What is immigration status of the communities that you see? (documented/undocumented, economic migrants, refugees, stateless people)
3. What are the demographic characteristics of the migrant workers you see? (male/female, occupation, country of origin)

**Open questions**

1. Do you have difficulty communicating with migrant workers? How do you overcome this?
2. What are the common conditions that migrant workers present with?
3. Who pays for migrant’s health services? Migrant/ employer
4. Do healthcare services for migrant workers cost more than Malaysians?
5. What is your opinion of migrant workers knowledge/awareness on healthcare issues?
6. Can you tell me about healthcare policy and services available for migrant workers in Malaysia?

Prompts: awareness of services available for migrants, insurance schemes, and injury compensation schemes

1. Could you tell me about FOMEMA services?
2. What is your opinion of occupational health and safety measures taken by migrant workers and their employers?
3. Do you provide sexual reproductive health services for migrants? E.g. contraception
4. Do you have any suggestions for future improvement for migrant workers in healthcare?
